# Reduced intrinsic neural timescales in schizophrenia along posterior parietal and occipital areas

**DOI:** 10.1038/s41537-021-00184-x

**Published:** 2021-11-22

**Authors:** Lavinia Carmen Uscătescu, Sarah Said-Yürekli, Lisa Kronbichler, Renate Stelzig-Schöler, Brandy-Gale Pearce, Luise Antonia Reich, Stefanie Weber, Wolfgang Aichhorn, Martin Kronbichler

**Affiliations:** 1 Centre for Cognitive Neuroscience and Department of Psychology, University of Salzburg, Salzburg, Austria; 2 Neuroscience Institute, Christian-Doppler Medical Centre, Paracelsus Medical University, Salzburg, Austria; 3 Department of Psychiatry, Psychotherapy and Psychosomatics, Christian-Doppler Medical Centre, Paracelsus Medical University, Salzburg, Austria; 4 University Medical Centre Hamburg—Eppendorf, Hamburg, Germany; 5 Present Address: Olin Neuropsychiatry Research Centre, Institute of Living, Hartford, CT USA

**Keywords:** Schizophrenia, Biomarkers

## Abstract

We computed intrinsic neural timescales (INT) based on resting-state functional magnetic resonance imaging (rsfMRI) data of healthy controls (HC) and patients with schizophrenia spectrum disorder (SZ) from three independently collected samples. Five clusters showed decreased INT in SZ compared to HC in all three samples: right occipital fusiform gyrus (rOFG), left superior occipital gyrus (lSOG), right superior occipital gyrus (rSOG), left lateral occipital cortex (lLOC) and right postcentral gyrus (rPG). In other words, it appears that sensory information in visual and posterior parietal areas is stored for reduced lengths of time in SZ compared to HC. Finally, we found that symptom severity appears to modulate INT of these areas in SZ.

## Introduction

Schizophrenia (SZ) is a psychiatric disorder diagnosed in ~1% of the world’s population^1^. It is characterised by negative (e.g., disorganised thoughts and language, attention and memory deficits) and positive (e.g., hallucinations and delusions) symptoms. Of high relevance to SZ pathology are the visual, auditory and sensorimotor areas. The dysconnectivity and disintegration of primary sensory areas have been proposed to underlie higher cognitive dysfunctions in SZ^2–4^ and have been shown to be predictive of disease severity^5–9^. For example, increased connectivity between early and late visual areas has been linked to mood induction in a compensatory manner in SZ^10^. Furthermore, cognitive control deficits in SZ patients have been linked to hyperconnectivity within the auditory, sensorimotor and posterior parietal cortex^11^. The thalamus, involved in sensory gating deficits in SZ, has also been shown to be hyperconnected to sensorimotor areas^12^, and its increased connectivity to the middle temporal gyrus has been positively related to the presence of hallucinations and delusions^13^. Finally, connectivity alterations in somatosensory areas have also been shown to be good predictors of patient classification^14^.

In later years, there has been a greater emphasis on characterising neuropsychiatric disorders in terms of trans-diagnostic, as opposed to categorical symptoms. One such symptom reflects sensory processing deficits^15,16^, which comprise responding to, processing and organising sensory information^17^. Sensory deficits have been found to characterise several neuropsychiatric disorders, such as SZ^18,19^ and autism spectrum disorders^20,21^ (ASD). True to its trans-diagnostic potential, when comparing ASD and SZ directly, these deficits have been found to be phenotypically similar in both disorders^22,23^.

Most neuroimaging research deals with analysing static relationships^24^ between functional neural components. However, this can only offer limited insight into brain health and disease, since brain activity is essentially dynamic. Consequently, a range of time series analyses directed at characterising the temporal changes in brain activity have been developed in recent years. One such approach is to assess how long information is stored in various neural areas. This duration is known as temporal receptive field, intrinsic neural timescales (INT), or temporal receptive window.

A hierarchical organisation of INT across the primate cortex has been initially noted based on spike count^25^, with sensory areas displaying shorter INT compared to frontal ones. Human neuroimaging studies on healthy populations have confirmed a similar hierarchical organisation, with longer INT in frontal and parietal compared to sensory areas^26,27^. This has been argued to form the basis of a functional hierarchy in the brain^28,29^ that enables sensory areas to register fast environmental changes^30^ and cognitive areas to integrate and analyse sensory input^31–33^. This functional hierarchy has a practical relevance for both localised and distributed neural activity, as shown by Ito, Hearne and Cole^34^. These authors showed that regions with faster INT during resting state displayed strong activations and decreased functional connectivity during task states. In addition, Fallon et al.^35^ further showed that increased INT correlated with increased structural connectivity, thus expanding on the practical implications of assessing cortical temporal dynamics. This intrinsic hierarchical functional organisation of brain activity and its alterations can improve the state of our current knowledge on how and where information processing breaks down in the healthy, but mostly in the dysfunctional brain.

This functional hierarchy has been shown to be altered in SZ. For example, Wengler et al.^36^ found reduced INT at the whole-brain level in SZ compared to HC, and showed that INT reduction in auditory areas appeared to be modulated by hallucination and delusion severity. We were therefore interested to see how well this pattern is replicable across independent SZ samples and whether symptom severity plays a modulatory role. For this purpose, rsfMRI data has been analysed along the lines of Watanabe, Rees and Masuda^37^. Replicability can be however dramatically compromised if false positives are not controlled for. One source of spurious results in resting-state fMRI analyses are head motion artefacts, which are particularly frequent in clinical populations^38,39^. Since we analysed data collected from SZ patients, this was a concern which we sought to address, therefore we analysed the INT-group differences both before and after eliminating framewise displacement outliers in all three SZ samples.

## Results

First, we ensured that sex and age did not differ significantly between SZ and HC in either of the three samples. For the in-house dataset, as it was all-male, we only checked that the two groups were age-balanced (mean (SD) of SZ = 26.26 (4.83); mean (SD) of HC = 25.1 (4.33); *t* (44.494) = −0.91523, *P* = 0.365). The COBRE sample was equally balanced for both sex (*χ*^2^(1) = 2.033, *P* = 0.134) and age (mean (SD) of SZ = 38.2 (13.9); mean (SD) of HC = 35.8 (11.6); *t* (138.07) = −1.105, *P* = 0.271). The UCLANP sample was equally sex (*χ*^2^(1) = 0.267, *P* = 0.61) and age (mean (SD) of SZ = 36.46 (8.88); mean (SD) of HC = 33.73 (9.1); *t* (106.31) = −1.606, *P* = 0.111) balanced.

### Voxel-wise exploratory results

**Fig. 1:**
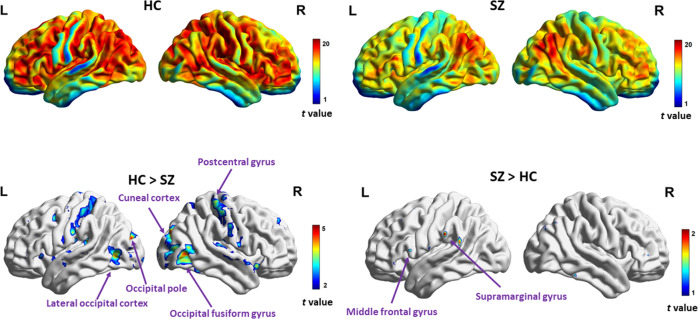
Mass-univariate exploratory analysis (*P* uncorr. <0.001) of INT within and between SZ and HC of the COBRE dataset. In the top left and right, we observe the expected pattern of increased INT in frontal and parietal areas in both groups. The lower left panel depicts the areas that show longer INT in HC compared to SZ, namely: the right postcentral gyrus, the right occipital fusiform gyrus and the right cuneal cortex, the left occipital pole and the left lateral occipital cortex. The lower right panel depicts the areas that show longer INT in SZ compared to HC, namely the left supramarginal and middle frontal gyrus.

Exploratory mass-univariate *t* tests were first performed in COBRE, to compare the INT index between HC and SZ. First, the expected pattern of increased INT^29^ (Lerner et al.) in frontal and parietal areas, and decreased INT in sensory areas was also confirmed in both HC and SZ (see Fig. 1 below, top left and right panels). A pattern of decreased INT in SZ compared to HC was observed in bilateral postcentral gyrus and occipital areas (see Fig. 1 bottom left panel and Table 1 below). Very few areas displayed increased INT in SZ compared to HC, in the supramarginal and inferior frontal gyrus (see Fig. 1 below, bottom right panel).

**Table 1:** Mean and standard deviations of symptom severity in all three patient samples.

	COBRE	INHOUSE	UCLANP
PANSS			
Positive	14.96 (4.83)	14.00 (5.72)	
Negative	14.53 (4.83)	15.48 (7.02)	
SANS			
Affective blunting			1.26 (1.31)
Alogia			0.98 (1.51)
Avolition			2.72 (1.51)
Anhedonia			2.28 (1.50)
Attention			2.16 (1.36)
SAPS			
Hallucinations			2.30 (1.75)
Delusions			2.54 (1.47)
Bizarre behaviour			0.96 (1.36)
Positive formal thought behaviour			1.60 (1.51)

### Region of interest (ROI) identification

Based on the mass-univariate results from the COBRE dataset, prior to FD outlier elimination, we selected the significant clusters and thus extracted the five ROIs (see Table 2 below) which were used in the subsequent cluster-level analyses. In order to avoid double-dipping, we excluded the COBRE dataset from the first set of ROI level group comparisons (i.e, prior to FD outlier elimination). We included it again when we re-ran our analyses post FD outlier elimination.

**Table 2:** Voxel-wise INT-group differences.

Anatomical label	Abbreviation	MNI coordinates			Cluster size	*T* value (FDR corr.)
HC > SZ		*X*	*Y*	*Z*		
Right occipital fusiform gyrus	rOFG	27	−88	−14	122	4.34
Left superior occipital gyrus	lSOG	−15	−91	19	136	5.39
Right superior occipital gyrus	rSOG	18	−79	25	452	5.04
Left lateral occipital cortex	lLOC	−42	−70	1	87	4.19
Right postcentral gyrus	rPG	42	−31	64	61	4.61

### Group differences in INT duration between HC and SZ

**Fig. 2:**
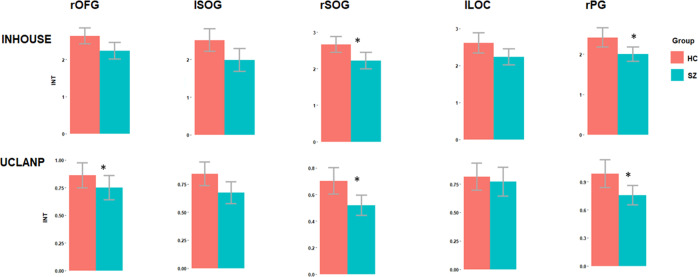
Group differences in intrinsic neural timescales (INT) per sample and cluster, prior to FD outlier elimination. Bonferroni-corrected significant differences are marked. Red colour represents HC, while blue represents SZ. The asterisk indicates a *P* < 0.05 significance level.

Following ROI identification, we proceeded to analyse group differences in INT duration between HC and SZ within each of the five ROIs, for both the in-house and the UCLANP samples. Welch two-samples *t* tests were used for this purpose, as implemented in the R software. A consistent pattern of reduced INT in SZ compared to HC was found in all ROIs, and replicated in both samples. Within the in-house dataset, significantly increased INT durations in HC compared to SZ were found in the rSOG (mean (SD) of INT in HC = 2.66 (0.61), mean (SD) of INT in SZ = 2.22 (0.55), *t* (54.19) = 2.86, *P* Bonf. = 0.03, Hedge’s g = 0.74) and the rPG (mean (SD) of INT in HC = 2.42 (0.67), mean (SD) of INT in SZ = 2 (0.43), *t* (54.74) = 2.87, *P* Bonf. = 0.03, Hedge’s g = 0.71). In UCLANP, significantly longer INT were found in HC compared to SZ in the rOFG (mean (SD) of INT in HC = 0.84 (0.42), mean (SD) of INT in SZ = 0.67 (0.34), *t* (110.92) = 2.37, *P* Bonf. = 0.05, Hedge’s g = 0.44), the rSOG (mean (SD) of INT in HC = 0. 7(0.39), mean (SD) of INT in SZ = 0.52 (0.27), *t* (108.46) = 2.95, *P* Bonf. = 0.05, Hedge’s g = 0.53) and the rPG (mean (SD) of INT in HC = 0.99 (0.59), mean (SD) of INT in SZ = 0.76 (0.36), *t* (105.34) = 2.54, *P* Bonf. = 0.03, Hedge’s g = 0.46). These results are illustrated in Fig. 2 below and summarised in Supplementary Table 1.

### Relationship between symptom severity and INT

We also explored the relationship between symptom severity and INT of the five clusters, in the three patient samples. We found no significant relationship between INT duration in any of the five clusters and symptom severity in our in-house sample (*r* < 0.21, *P* > 0.31) and in COBRE (*r* < 0.24, *P* > 0.22). In the UCLANP samples, we found a significant negative correlation between the INT duration of the Right occipital fusiform gyrus and positive formal thought disorder (*r* = −0.3, *P* = 0.03).

### Elimination of outliers with extreme head motion artefacts

**Fig. 3:**
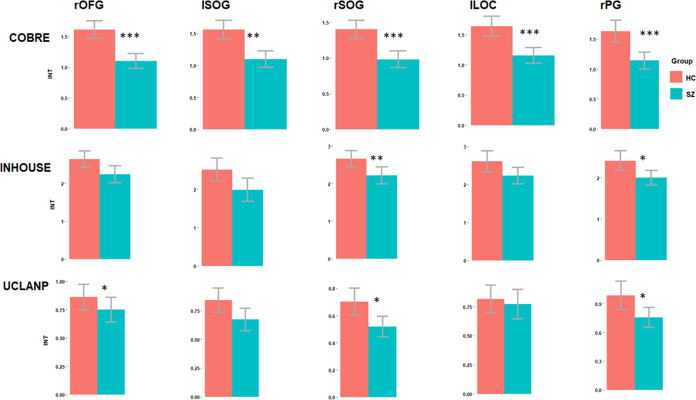
Group differences in intrinsic neural timescales (INT) per sample and cluster, after FD outlier elimination. Bonferroni-corrected significant differences are marked. Red colour represents HC, while blue represents SZ. The asterisk indicates a *P* < 0.05 significance level.

Due to concerns regarding the false-positive rate which might be driven upwards by motion artefacts, we re-analysed the INT-group differences after FD outlier elimination. The previously observed pattern of decreased INT in SZ compared to HC is preserved across all clusters and samples, though only the group differences for rSOG and rPG were consistently statistically significant in all three datasets. In COBRE, significantly longer INT in HC compared to SZ were found in all five clusters: rOFG (mean (SD) of INT in HC = 1.56 (0.57), mean (SD) of INT in SZ = 1.16 (0.49), *t* (111.6) = 4.1, *P* Bonf. <0.001, Hedge’s g = 0.74), lSOG (mean (SD) of INT in HC = 1.53 (0.64), mean (SD) of INT in SZ = 1.17 (0.55), *t* (111.6) = 3.33, *P* Bonf. = 0.005, Hedge’s g = 0.61), rSOG (mean (SD) of INT in HC = 1.41 (0.54), mean (SD) of INT in SZ = 1.01 (0.46), *t* (111.27) = 4.32, *P* Bonf. <0.001, Hedge’s g = 0.8), lLOC (mean (SD) of INT in HC = 1.62 (0.68), mean (SD) of INT in SZ = 1.19 (0.54), *t* (108.79) = 3.81, *P* Bonf. <0.001, Hedge’s g = 0.71) and rPG (mean (SD) of INT in HC = 1.63 (0.69), mean (SD) of INT in SZ = 1.14 (0.53), *t* (107.5) = 4.31, *P* Bonf. <0.001, Hedge’s g = 0.8). In the in-house sample, significantly longer INT in HC compared to SZ were found in rSOG (mean (SD) of INT in HC = 2.59 (0.43), mean (SD) of INT in SZ = 2.1 (0.44), *t* (26.29) = 2.14, *P* Bonf. = 0.005, Hedge’s g = 1.15) and rPG (mean (SD) of INT in HC = 2.45 (0.65), mean (SD) of INT in SZ = 1.9 (0.45), *t* (28.23) = 2.89, *P* Bonf. = 0.04, Hedge’s g = 0.96). In UCLANP, significantly longer INT in HC compared to SZ were found in rOFG (mean (SD) of INT in HC = 0.84 (0.42), mean (SD) of INT in SZ = 0.67 (0.34), *t* (107) = 2.32, *P* Bonf. = 0.05, Hedge’s g = 0.44), rSOG (mean (SD) of INT in HC = 0.7 (0.4), mean (SD) of INT in SZ = 0.51 (0.27), *t* (103.21) = 2.88, *P* Bonf. = 0.01, Hedge’s g = 0.53) and rPG (mean (SD) of INT in HC = 0.97 (0.58), mean (SD) of INT in SZ = 0.75 (0.37), *t* (101.34) = 2.39, *P* Bonf. = 0.05, Hedge’s g = 0.44). These results are illustrated in Fig. 3 below and summarised in Supplementary Table 2.

**Fig. 4:**
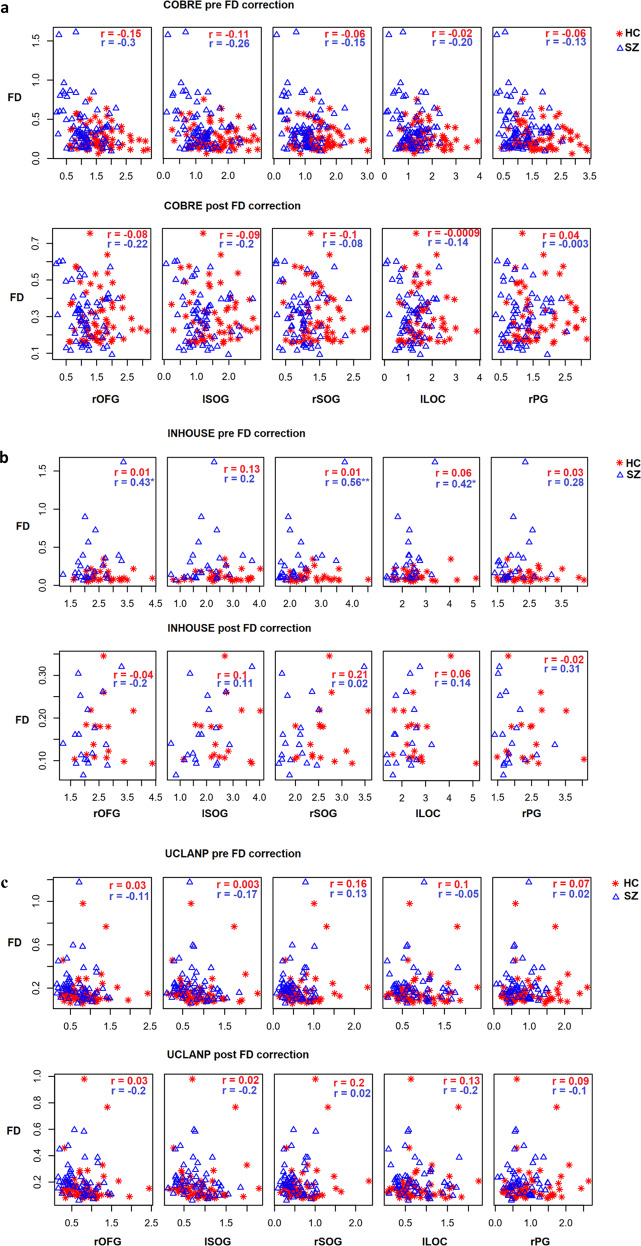
Correlations between framewise displacement (FD) and intrinsic neural timescales (INT), before and after FD outlier elimination, per sample and cluster. COBRE in panel **a**, INHOUSE in panel **b**, and UCLANP in panel **c**. Red star symbols represent correlations between FD and INT in HC, while blue triangle symbols represent correlations between FD and INT in SZ. Correlation coefficients are written in red for HC and blue for SZ. The asterisk indicates a *P* < 0.05 significance level.

Finally, to ensure that INT-group differences are not due to motion artefacts, we further explored the relationship between FD and INT. In COBRE and UCLANP there were no significant correlations between INT duration and FD, neither before nor after FD outlier elimination (see Fig. 4 below). In the in-house SZ sample, there were three significant positive correlations between FD and INT duration in the rOFG (*r* = 0.43, *P* = 0.03, *P* Bonf. = 0.2), rSOG (*r* = 0.56, *P* = 0.004, *P* Bonf. = 0.02) and lLOC (*r* = 0.42, *P* = .04, *P* Bonf. = 0.2). However, this relationship became non-significant after FD outlier elimination. Thus, even if motion artefacts might have had an initial influence on group differences, we believe that this was removed in the second step, by eliminating extreme FD values.

### Relationship between symptom severity and INT after FD outlier elimination

We analysed the relationship between symptom severity and INT of the five clusters, in the three patient samples, following FD outlier elimination. No significant correlations were found in the in-house sample (*r* < −0.41, *P* > 0.1). In the UCLANP sample, we found a significant negative correlation between the INT of the rOFG and positive formal thought disorder (*r* = –0.3, *P* = 0.03, *P* Bonf. = 1). In COBRE, one significant negative correlation was found between the INT of the lSOG and the general psychopathology factor (*r* = −0.4, *P* = 0.04, *P* Bonf. = 0.62).

### Sex differences in INT before FD outlier elimination

Sex differences were analysed for the two datasets that contained both male and female participants, i.e., COBRE and UCLANP. This was done both before and after eliminating FD outliers.

#### COBRE

The main effect of sex was significant for rSOG (*F* = 6.914, *P* = 0.009) and rPG (*F* = 4.756, *P* = 0.031). We subsequently ran an ANCOVA analysis to check for group differences in INT while controlling for sex. All group differences remained significant after controlling for sex. Results are summarised in Supplementary Table 3.

#### UCLANP

The main effect of sex was significant for rOFG (*F* = 9.133, *P* = 0.003), lSOG (*F* = 7.881, *P* = 0.006), rSOG (*F* = 5.566, *P* = 0.02), lLOC (*F* = 8.448, *P* = 0.004) and rPG (*F* = 5.95, *P* = 0.02). We subsequently ran an ANCOVA analysis to check for group differences in INT while controlling for sex. Group differences for rOFG, rSOG, and rPG remained significant after controlling for sex. Results are summarised in Supplementary Table 3.

### Sex differences in INT after FD outlier elimination

#### COBRE

After eliminating FD outliers, the main effect of sex remained significant for rPG (*F* = 4.664, *P* = 0.03). We subsequently ran an ANCOVA analysis to check for group differences in INT while controlling for sex. All group differences remained significant after controlling for sex. Results are summarised in Supplementary Table 4.

#### UCLANP

The main effect of sex was significant for rOFG (*F* = 8.176, *P* = 0.005), lSOG (*F* = 7.724, *P* = 0.006), rSOG (*F* = 5.716, *P* = 0.02), lLOC (*F* = 7.505, *P* = 0.007) and rPG (*F* = 4.537, *P* = 0.04). We subsequently ran an ANCOVA analysis to check for group differences in INT while controlling for sex. Group differences for rOFG, lSOG, rSOG and rPG remained significant after controlling for sex. Results are summarised in Supplementary Table 4.

#### Medication and INT

The potential impact of medication (as reflected by the Chlorpromazine/CPZ equivalent dosage) on INT was analysed in all three patient samples, before and after FD outlier elimination (COBRE: mean CPZ before = 499.18 ± 1081.9, mean CPZ after = 359.11 ± 311.96; UCLANP: mean CPZ before = 879.29 ± 1251.7, mean CPZ after = 898.54 ± 1260.94; INHOUSE: mean CPZ before = 476.63 ± 232, mean CPZ after = ). The CPZ equivalent was calculated according to Gardner et al.^40^, using the R package “chlorpromazineR” developed by Brown, Shah, & Kim^41^.

#### INHOUSE

No significant main effect of medication dosage was found prior to FD outlier elimination for either of the five ROIs (*P* > 0.1). The same was true after eliminating FD outliers (*P* > 0.2).

#### COBRE

One significant main effect of medication dosage on the INT of lSOG prior to FD outlier elimination was found (*F* = 4.373, *P* = 0.04). Following outlier elimination, medication showed no significant main effect on the INT of either ROI (*P* > 0.7).

#### UCLANP

No significant main effect of medication dosage was found prior to FD outlier elimination for either of the five ROIs (*P* > 0.2). The same was true after eliminating FD outliers (*P* > 0.2).

## Discussion

In this paper, we present a replication of intrinsic neural timescales (INT) patterns in three independent samples of patients with schizophrenia (SZ) and matched healthy controls (HC). Our main goal was to assess to which extent INT findings can be reliably replicated across independent samples. We believe this to be a crucial step, as it has been shown that different data acquisition settings constitute a major obstacle in achieving replicability^42^. We pre-processed and analysed all three samples identically and we were able to show that the pattern of reduced INT in SZ compared to HC also generalises well across independently acquired samples, thus being robust to differences in acquisition protocols.

Motion artefacts were another concern that we sought to address, as they tend to occur frequently in clinical samples and often lead to spurious results^38,39^. We, therefore, assessed the INT patterns before and after FD outlier elimination, and our results were significant in both instances. In addition, we also checked whether a relationship between FD and INT might have led to false positives. In most cases, this relationship was not significant, even before eliminating FD outliers. In the few cases where there was a positive and significant relationship between FD and INT, this effect disappeared once we eliminated FD outliers. The pattern of reduced INT in SZ compared to HC was however invariably preserved. We were therefore satisfied that the results we observed reflected real and replicable group differences and not spurious results driven by motion artefacts.

One mechanistic interpretation with respect to the reduced INT in SZ was previously offered by Wengler et al.^36^. Following a series of simulations, these authors suggest that a reduction in the excitation–inhibition (E/I) ratio could account for the global, brain-level INT reduction in SZ compared to HC. Computational studies have also previously linked long-range autocorrelation fluctuations to the E/I ratio^43^. This mechanism appears to be supported by clinical findings as well, as similar E/I ratio imbalances have been found, for example, in both ASD and SZ^44^. Given the overlap in sensory impairments between these two disorders, it is reasonable to propose that the INT patterns can be used as a trans-diagnostic biomarker bridging ASD and SZ^45^, and capturing underlying E/I imbalances in sensory areas.

We also assessed the relationship between INT and symptom severity and found a negative significant correlation between INT in the rOFG and positive formal thought disorder in the UCLANP sample. In other words, it appears that increased severity of this symptom is associated with increased excitation/inhibition (EI) ratio (hence decreased INT) in the rOFG. Previously, Wengler et al.^36^ looked at the relationship between hallucination and delusion severity in SZ and INT of auditory and visual areas. While they found such an association for the auditory system, they did not find any for the visual system. This may be explained by the different approaches in parcellation and ROI identification between our study and that of Wengler et al.^36^. While Wengler et al.^36^ opted for anatomically defined parcels, we opted for a data-driven functional ROI identification based on group differences in the COBRE dataset. We believe that the difference in acquisition parameters of the different datasets (i.e., the Human Connectome Project in Wengler et al.^36^, and the COBRE, UCLANP and in-house datasets of our study) had little influence over our result differences since we already showed that INT patterns generalise well across different datasets.

An alternative explanatory mechanism that we propose for the reduced INT similarities between the two disorders could be sensory gating impairments, frequently documented in SZ and linked to thalamus and hypothalamus impairments^46–51^. Furthermore, Raut, Snyder and Raichle^52^ showed that the hierarchical cortical INT patterns are reflected within the thalamus, with INT increasing along a ventrolateral to the dorsomedial axis, essentially from lower- to higher-order nuclei. Based on the evidence listed here, it appears that thalamic mediated sensory gating could lead to the observed INT-group differences. However, further analyses linking the connectivity between the thalamus, hippocampus, frontal and sensory areas^53,54^ to the INT of sensory areas could provide the necessary evidence in favour of this proposed mechanism. As sensory integration alterations have been proposed to be a promising trans-diagnostic biomarker^15^, we argue that INT analysis offers a suitable approach, as it can reveal the temporal fluctuations of thalamocortical and cortico-cortical connections, arguably capturing a more accurate representation of neural activity.

Finally, we would like to mention two main limitations of our current study. First, due to our patient samples being fully medicated, we were unable to investigate whether medication had any definite impact on INT in SZ. Second, no structural connectivity analyses were performed as part of the current study, though these would be of great relevance especially with respect to the SZ patient population.

## Methods

### Participants

Three independently collected samples were used in the present study; (1) an in-house all-male dataset collected at the Centre for Cognitive Neurosciences in Salzburg, Austria; (2) an open-source dataset from the Center for Biomedical Research Excellence^54–57^ (COBRE; available at http://fcon_1000.projects.nitrc.org/indi/retro/cobre.html); (3) an open-source dataset from the UCLA Consortium for Neuropsychiatric Phenomics LA5c Study (UCLANP; available at https://www.openfmri.org/dataset/ds000030/). Age and sex differences were not significant between HC and SZ of all three samples.

The in-house sample consisted of 25 all-male patients (age mean and SD: 26.26 (4.83)) recruited at the Department of Psychiatry, Psychotherapy and Psychosomatics at the Christian-Doppler Medical Centre in Salzburg, Austria who had received a formal ICD-10 diagnosis in the schizophrenia spectrum group (F20) or the schizoaffective disorders spectrum group (F25). All of them provided written informed consent prior to their participation in the current study. At the time of scanning, patients were medicated and clinically stable, with mild symptom severity, as assessed with the Positive and Negative Syndrome Scale^58^ (PANSS). Two of the patients did not complete the PANSS assessment but did complete the resting-state scanning session. Thirty-one age and education matched control participants (age mean and SD: 25.10 (4.33)) were recruited and screened for mental and physical health (via a standardised anamnesis procedure) and were excluded if they reported a history of mental or neurological disorder or a family history of psychiatric disorders. All participants provided written informed consent before taking part in the study. The study was approved by the Ethics Board of the University of Salzburg. More details about the recruitment and assessment of the participants included in the present study can be found in Kronbichler et al.^59^. Functional imaging data were acquired on a Siemens Magnetom Trio 3 T scanner (Siemens AG, Erlangen, Germany) using a 32-channel head coil. Functional images were acquired with a T2*-weighted gradient-echo EPI sequence (TR 2250 ms, TE 30 ms, matrix 64 × 64 mm, FOV 192 mm, flip angle 70°). Thirty-six slices with a slice thickness of 3 mm and a slice gap of 0.3 mm were acquired within the TR. Scanning was completed over two sessions with 321 scans per session. Finally, a gradient-echo field map (TR 488 ms, TE 1 = 4.49 ms, TE 2 = 6.95 ms) and a high-resolution (1 × 1 × 1 mm) structural scan with a T1-weighted MPRAGE sequence were also acquired.

Seventy-two SZ (58 males; age mean and SD: 38.17 (13.98)) and seventy-four HC (51 males; age mean and SD: 35.82 (11.58)) from the COBRE open-source dataset were included in this study. All participants were screened for a history of neurological disorders, mental retardation, and severe head trauma with more than 5 min loss of consciousness, substance abuse or dependence within the last 12 months. Clinical diagnosis was established using the Structured Clinical Interview used for DSM Disorders^60^ (SCID-IV). A multi-echo MPRAGE (MEMPR) sequence was ran with the following parameters: TR/TE/TI = 2530/[1.64, 3.5, 5.36, 7.22, 9.08]/900 ms, flip angle = 7°, FOV = 256 × 256 mm, slab thickness = 176 mm, matrix = 256 × 256 × 176, Voxel size = 1 × 1 × 1 mm, number of echos = 5, pixel bandwidth = 650 Hz, total scan time = 6 min. Resting-state data were collected with single-shot full k-space echo-planar imaging (EPI) with ramp sampling correction using the anterior-to-posterior commissural line as a reference (TR: 2 s, TE: 29 ms, matrix size: 64 × 64, 32 slices, voxel size: 3 × 3 × 4 mm^3^).

Fifty SZ (38 males; age mean and SD: 36.46 (8.88)) and sixty-three (44 males; age mean and SD: 33.73 (9.1)) HC were included from the UCLA Consortium for Neuropsychiatric Phenomics LA5c Study. Participants were screened for neurological disease and major mental illness, history of head injury with loss of consciousness, use of psychoactive medications, and substance dependence within 6 months prior to testing. Self-reported history of psychopathology was assessed with the SCID-IV^60^. Urinalysis was used to screen for drugs of abuse (cannabis, amphetamine, opioids, cocaine, benzodiazepines) on the day of testing and participants were excluded if their results were positive. Neuroimaging data were acquired on a 3 T Siemens Trio scanner. Functional MRI data were collected with a T2*-weighted echo-planar imaging (EPI) sequence with the following parameters: slice thickness = 4 mm, 34 slices, TR = 2 s, TE = 30 ms, flip angle = 90°, matrix = 64 × 64, FOV = 192 mm. A T1-weighted high-resolution anatomical scan (MPRAGE) was collected with the following parameter: slice thickness = 1 mm, 176 slices, TR = 1.9 s, TE = 2.26 ms, matrix = 256 × 256, FOV = 250 mm. The eyes open resting-state fMRI session lasted for 304 s.

Symptom severity was assessed using The Scale for the Assessment of Negative Symptoms^61^ (SANS) and the Scale for the Assessment of Positive Symptoms^62^ (SAPS) for the UCLANP sample, and the PANSS for the COBRE and the in-house samples. Means and standard deviations of symptom severity measures for all three patient samples are given in Table 1.

### Neuroimaging data pre-processing

The fMRI data were pre-processed and analysed using SPM12 (Wellcome Trust Centre for Neuroimaging, London, UK; code available at https://github.com/spm/spm12), while all the other statistical analyses were performed in R 5.2^63^. Functional scans were realigned, de-spiked, unwarped, corrected for geometric distortions, and slice time corrected. They were also normalised to MNI space and co-registered to the corresponding skull-stripped structural images, and afterwards resampled to 3 × 3 × 3 mm voxels and smoothed with a 6 mm FWHM Gaussian kernel. Motion correction was performed using ICA-AROMA^64^ (available at: http://fsl.fmrib.ox.ac.uk/fsl/fslwiki/OtherSoftware), and the resulting non-aggressively corrected resting-state time series were used for computing the INT.

### Analysis

Following the INT analysis of Watanabe et al.^37^, the autocorrelation function was calculated for each voxel at incremental time lags until the autocorrelation function value became negative for the first time. The positive autocorrelation values were then summed up. The resulting sum was then multiplied by the repetition time (TR) to account for temporal resolution differences between the three samples. An index of the INT was thus obtained.

Next, using the COBRE dataset, we identified group differences in INT duration between HC and SZ via voxel-wise analysis. We isolated five clusters which we then used as masks to extract INT duration from those specific locations in HC and SZ from the in-house and the UCLANP datasets. This extraction was performed using the REX toolbox. Individual INT values were then exported and subsequently analysed using the R 5.2^63^ software. Our choice in using the COBRE as a starting point was twofold; it is the most widely used within the community, and it allowed us to include the largest number of participants.

As previous concerns have been raised regarding the possibility that head motion artefacts can cause false-positive results, we re-analysed INT duration group differences after eliminating motion artefacts outliers. These artefacts (i.e., framewise displacement parameters; FD) were identified using the FSL library^65^. Group differences in FD between HC and SZ were minimised in two steps. First, the SZ with the largest FD values were gradually eliminated until the FD group difference was no longer significant (i.e., *P* > 0.05). Then, the HC with the smallest FD values were gradually eliminated until the mean FD values of the two groups were as similar as possible (*P* ≈ 1).

### Reporting summary

Further information on research design is available in the Nature Research Reporting Summary linked to this article.

## Supplementary information

Supplementary Information

REPORTING SUMMARY

## Data Availability

The COBRE and UCLANP datasets are freely available online. The COBRE can be downloaded from http://fcon_1000.projects.nitrc.org/indi/retro/cobre.html. The UCLANP can be downloaded from https://exhibits.stanford.edu/data/catalog/mg599hw5271. The in-house dataset can be obtained upon request. The R code that we used for analysing this data will also be made available upon request.
