# Supplementary material for: Reduced intrinsic neural timescales in schizophrenia along posterior parietal and occipital areas
Source: NPJ Schizophr. 2021 Nov 22;7:55. doi: 10.1038/s41537-021-00184-x (PMC8608811; doi:10.1038/s41537-021-00184-x)
Supplement: Supplementary file 1 — Supplementary Information [file 41537_2021_184_MOESM1_ESM.docx]

**Supplementary Table 1.** INT group differences per sample and cluster, *before FD outlier elimination*. Both uncorrected and Bonferroni corrected p values are given. Due to the HC and SZ sample sizes not being identical, Hedge’s g was preferred as an effect size estimator. The HC > SZ comparison was assessed using one-sided Welch two-samples t tests.

|  | Mean (SD) INT | | HC > SZ | | | | |
| --- | --- | --- | --- | --- | --- | --- | --- |
|  | HC SZ |  | t | df | *p (uncorr)* | *p  (Bonf. Corr)* | *Hedge’s g* |
|  |  |  |  |  |  |  |  |
| *INHOUSE* |  |  |  |  |  |  |  |
| rOFG  lSOG | 2.64 (0.61) 2.24 (0.54)  2.51 (0.84) 1.98 (0.73) |  | 2.63  2.55 | 54.43  54.8 | .011  .014 | .055  .07 | 0.68  0.66 |
| rSOG | 2.66 (0.61) 2.22 (0.55) |  | 2.86 | 54.19 | .006 | .03 | 0.74 |
| lLOC  rPG | 2.61 (0.77) 2.34 (0.52)  2.42 (0.67) 2.0 (0.43) |  | 2.23  2.87 | 55.37  54.74 | .03  .006 | .15  .03 | 0.55  0.71 |
| *UCLANP* |  |  |  |  |  |  |  |
| rOFG | 0.84 (0.42) 0.67 (0.34) |  | 2.37 | 110.92 | .01 | .05 | 0.44 |
| lSOG | 0.86 (0.44) 0.75 (0.39) |  | 1.42 | 110.08 | .08 | .4 | 0.26 |
| rSOG | 0.7 (0.39) 0.52 (0.27) |  | 2.95 | 108.46 | .01 | .05 | 0.53 |
| lLOC | 0.81 (0.47) 0.77 (0.44) |  | 0.51 | 107.85 | .31 | 1 | 0.1 |
| rPG | 0.99 (0.59) 0.76 (0.36) |  | 2.54 | 105.34 | .006 | .03 | 0.46 |
|  |  |  |  |  |  |  |  |

**Supplementary Table 2.** INT group differences per sample and cluster, *after FD outlier elimination.* Both uncorrected and Bonferroni corrected p values are given. Due to the HC and SZ sample sizes not being identical, Hedge’s g was preferred as an effect size estimator. The HC > SZ comparison was assessed using one-sided Welch two-sample t tests.

|  | Mean (SD) INT | | HC > SZ | | | | |
| --- | --- | --- | --- | --- | --- | --- | --- |
|  | HC SZ |  | t | df | *p (uncorr)* | *p  (Bonf. Corr)* | *Hedge’s g* |
|  |  |  |  |  |  |  |  |
| *COBRE* |  |  |  |  |  |  |  |
| rOFG  lSOG  rSOG  lLOC  rPG  INHOUSE | 1.56 (0.57) 1.16 (0.49)  1.53 (0.64) 1.17 (0.55)  1.41 (0.54) 1.01 (0.46)  1.62 (0.68) 1.19 (0.54)  1.63 (0.69) 1.14 (0.53) |  | 4.1  3.33  4.32  3.81  4.31 | 111.6  111.6  111.27  108.79  107.5 | < .001  .001  < .001  < .001  < .001 | < .001  .005  < .001  < .001  < .001 | 0.74  0.61  0.8  0.71  0.8 |
| rOFG  lSOG  rSOG  lLOC  rPG | 2.65 (0.66) 2.12 (0.5)  2.6 (0.77) 1.89 (0.77)  2.59 (0.43) 2.1 (0.44)  2.66 (0.84) 2.15 (0.52)  2.45 (0.65) 1.9 (0.45) |  | 2.66  2.71  3.48  2.14  2.89 | 29.55  32.87  32.98  26.29  28.23 | .012  .011  .001  .04  .007 | .06  .06  .005  .2  .04 | 0.89  0.9  1.15  0.72  0.96 |
| UCLANP |  |  |  |  |  |  |  |
| rOFG  lSOG  rSOG lLOC  rPG | 0.84 (0.42) 0.67 (0.34)  0.85 (0.44) 0.85 (0.39)  0.7 (0.4) 0.51 (0.27)  0.8 (0.47) 0.77 (0.45)  0.97 (0.58) 0.75 (0.37) |  | 2.32  1.23  2.88  .35  2.39 | 107  106.14  103.21  104.61  101.34 | .01  .11  .002  .36  .01 | .05  .6  .01  1  .05 | 0.44  0.23  0.53  0.1  0.44 |
|  |  |  |  |  |  |  |  |

**Supplementary Table 3.** Group differences in INT were analysed with ANCOVA using sex as covariate, prior to FD outlier elimination. The results of post-hoc analyses with Welch two sample t-tests with both uncorrected and Bonferroni corrected p values and Hedge’s g effect sizes are summarized.

|  | HC vs. SZ | | HC > SZ | | | | |
| --- | --- | --- | --- | --- | --- | --- | --- |
|  | F(1) *p* ηp2 |  | t | df | *p (uncorr)* | *p  (Bonf. Corr)* | *Hedge’s g* |
|  |  |  |  |  |  |  |  |
| *COBRE* |  |  |  |  |  |  |  |
| rOFG  lSOG | 34.3 .009 0.2  26 .000 0.15 |  | 5.45  4.74 | 141.28  142.65 | .000  .000 | .000  .000 | 0.9  0.78 |
| rSOG | 30.6 .000 0.18 |  | 4.9 | 143.48 | .000 | .000 | 0.81 |
| lLOC  rPG | 24.17 .000 0.15  36 .000 0.2 |  | 4.61  5.47 | 137.89  135.25 | .000  .000 | .000  .000 | 0.76  0.9 |
| *UCLANP* |  |  |  |  |  |  |  |
| rOFG | 6.93 .01 0.1 |  | 2.37 | 110.92 | .01 | .05 | 0.44 |
| lSOG | 2.71 .1 0.02 |  | 1.42 | 110.08 | .08 | .39 | 0.26 |
| rSOG | 9.48 .003 0.08 |  | 2.95 | 108.46 | .002 | .01 | 0.53 |
| lLOC | 0.52 .47 0.01 |  | 0.51 | 107.85 | .31 | 1 | 0.1 |
| rPG | 1.67 .01 0.06 |  | 2.54 | 105.34 | .01 | .03 | 0.45 |
|  |  |  |  |  |  |  |  |

**Supplementary Table 4.** Group differences in INT were analysed with ANCOVA using sex as covariate, following FD outlier elimination. The results of post-hoc analyses with Welch two sample t-tests with both uncorrected and Bonferroni corrected p values and Hedge’s g effect sizes are summarized.

|  | HC vs. SZ | | HC > SZ | | | | |
| --- | --- | --- | --- | --- | --- | --- | --- |
|  | F(1) *p* ηp2 |  | t | df | *p (uncorr)* | *p  (Bonf. Corr)* | *Hedge’s g* |
|  |  |  |  |  |  |  |  |
| *COBRE* |  |  |  |  |  |  |  |
| rOFG  lSOG | 17.94 .000 0.14  12.3 .000 0.1 |  | 4.03  3.33 | 111.6  111.66 | .000  .000 | .000  .003 | 0.74  0.61 |
| rSOG | 5.33 .000 0.16 |  | 4.32 | 111.27 | .000 | .000 | 0.79 |
| lLOC  rPG | 16.36 .000 0.16  23 .000 0.17 |  | 3.81  4.31 | 108.79  107.5 | .000  .000 | .000  .000 | 0.7  0.79 |
| *UCLANP* |  |  |  |  |  |  |  |
| rOFG | 6.49 .01 0.06 |  | 2.32 | 107 | .01 | .05 | 0.44 |
| lSOG | 2.1 .16 0.02 |  | 1.23 | 106.14 | .11 | .55 | 0.23 |
| rSOG | 9 .003 0.1 |  | 2.88 | 103.21 | .002 | .01 | 0.53 |
| lLOC | 0.28 .6 0.003 |  | 0.35 | 104.61 | .36 | 1 | 0.1 |
| rPG | 6.14 .02 0.06 |  | 2.39 | 101.34 | .01 | .05 | 0.44 |
|  |  |  |  |  |  |  |  |
