# Supplementary material for: Reduced intrinsic neural timescales in schizophrenia along posterior parietal and occipital areas
Source: NPJ Schizophr. 2021 Nov 22;7:55. doi: 10.1038/s41537-021-00184-x (PMC8608811; doi:10.1038/s41537-021-00184-x)
Supplement: Supplementary file 2 — REPORTING SUMMARY [file 41537_2021_184_MOESM2_ESM.pdf]

nature portfolio

Corresponding author(s): Ms Lavinia Uscatescu

Last updated by author(s): Oct 17, 2021

Reporting Summary

Nature Portfolio wishes to improve the reproducibility of the work that we publish. This form provides structure for consistency and transparency in reporting. For further information on Nature Portfolio policies, see our Editorial Policies and the Editorial Policy Checklist.

Statistics

For all statistical analyses, confirm that the following items are present in the figure legend, table legend, main text, or Methods section.

n/a	Confirmed	
☐	☒	The exact sample size (*n*) for each experimental group/condition, given as a discrete number and unit of measurement
☐	☒	A statement on whether measurements were taken from distinct samples or whether the same sample was measured repeatedly
☐	☒	The statistical test(s) used AND whether they are one- or two-sided<br>*Only common tests should be described solely by name; describe more complex techniques in the Methods section.*
☐	☒	A description of all covariates tested
☐	☒	A description of any assumptions or corrections, such as tests of normality and adjustment for multiple comparisons
☐	☒	A full description of the statistical parameters including central tendency (e.g. means) or other basic estimates (e.g. regression coefficient) AND variation (e.g. standard deviation) or associated estimates of uncertainty (e.g. confidence intervals)
☐	☒	For null hypothesis testing, the test statistic (e.g. *F*, *t*, *r*) with confidence intervals, effect sizes, degrees of freedom and *P* value noted<br>*Give P values as exact values whenever suitable.*
☒	☐	For Bayesian analysis, information on the choice of priors and Markov chain Monte Carlo settings
☒	☐	For hierarchical and complex designs, identification of the appropriate level for tests and full reporting of outcomes
☐	☒	Estimates of effect sizes (e.g. Cohen's *d*, Pearson's *r*), indicating how they were calculated

Our web collection on statistics for biologists contains articles on many of the points above.

Software and code

Policy information about availability of computer code

Data collection	Data was collected either via pen and paper (e.g., in the case of self report scales), or via fMRI Siemens software. NO other software was used in collecting data.
Data analysis	Data preprocessing and the whole-brain analysis were performed using SPM12 and ICA-AROMA. The autocorrelation function was computed via a Matlab 2008b script. All other statistical analysis was performed using R.

For manuscripts utilizing custom algorithms or software that are central to the research but not yet described in published literature, software must be made available to editors and reviewers. We strongly encourage code deposition in a community repository (e.g. GitHub). See the Nature Portfolio guidelines for submitting code & software for further information.

Data

Policy information about availability of data

All manuscripts must include a data availability statement. This statement should provide the following information, where applicable:

- Accession codes, unique identifiers, or web links for publicly available datasets
- A description of any restrictions on data availability
- For clinical datasets or third party data, please ensure that the statement adheres to our policy

The COBRE and UCLANP datasets are freely available online. The COBRE can be downloaded from http://fcon_1000.projects.nitrc.org/indi/retro/cobre.html. The UCLANP can be downloaded from https://exhibits.stanford.edu/data/catalog/mg599hw5271. The inhouse dataset can be obtained upon request. The R code that we used for analysing this data will also be made available upon request.

Field-specific reporting

Please select the one below that is the best fit for your research. If you are not sure, read the appropriate sections before making your selection.

☒ Life sciences ☐ Behavioural & social sciences ☐ Ecological, evolutionary & environmental sciences

For a reference copy of the document with all sections, see nature.com/documents/nr-reporting-summary-flat.pdf

Life sciences study design

All studies must disclose on these points even when the disclosure is negative.

Sample size	INHOUSE sample: 56; COBRE: 132; UCLANP: 113
Data exclusions	No data were excluded.
Replication	WE replicate our results across three independent samples.
Randomization	Allocation was based on presence or absence of diagnosis, and all participants provided a resting state fMRI scan, so further randomization was not necessary in this study.
Blinding	Blinding was not necessary in this case.

Reporting for specific materials, systems and methods

We require information from authors about some types of materials, experimental systems and methods used in many studies. Here, indicate whether each material, system or method listed is relevant to your study. If you are not sure if a list item applies to your research, read the appropriate section before selecting a response.

Materials & experimental systems

n/a	Involved in the study	
☒	☐	Antibodies
☒	☐	Eukaryotic cell lines
☒	☐	Palaeontology and archaeology
☒	☐	Animals and other organisms
☐	☒	Human research participants
☒	☐	Clinical data
☒	☐	Dual use research of concern

Methods

n/a	Involved in the study	
☒	☐	ChIP-seq
☒	☐	Flow cytometry
☐	☒	MRI-based neuroimaging

Human research participants

Policy information about studies involving human research participants

Population characteristics	The in-house sample consisted of 25 all-male patients (Age mean and sd: 26.26 (4.83)). At the time of scanning, patients were medicated and clinically stable, with mild symptom severity, as assessed with PANSS (Kay, Fiszbein and Opler, 1987). Two of the patients did not complete the PANSS assessment but did complete the resting state scanning session. Thirty-one age and education matched control participants (Age mean and sd: 25.10 (4.33)) were recruited and screened for mental and physical health (via a standardized anamnesis procedure) and were excluded if they reported a history of mental or neurological disorder or a family history of psychiatric disorders.<br>Seventy-two SZ (58 males; Age mean and sd: 38.17 (13.98)) and seventy-four HC (51 males; Age mean and sd: 35.82 (11.58)) from the COBRE open source dataset were included in this study. All participants were screened for a history of neurological disorders, mental retardation, and severe head trauma with more than 5 minutes loss of consciousness, substance abuse or dependence within the last 12 months. Clinical diagnosis was established using the Structured Clinical Interview used for DSM Disorders (SCID).<br>Fifty SZ (38 males; Age mean and sd: 36.46 (8.88)) and sixty-three (44 males; Age mean and sd: 33.73 (9.1)) HC were included from the UCLA Consortium for Neuropsychiatric Phenomics LA5c Study. Participants were screened for neurological disease and major mental illness, history of head injury with loss of consciousness, use of psychoactive medications, and substance dependence within 6 months prior to testing. Self-reported history of psychopathology was assessed with the SCID-IV (First, Spitzer, Gibbon, & Williams, 1995). Urinalysis was used to screen for drugs of abuse (cannabis, amphetamine, opioids, cocaine, benzodiazepines) on the day of testing and participants were excluded if their results were positive.
Recruitment	The INHOUSE patients were recruited at the Department of Psychiatry, Psychotherapy and Psychosomatics at the Christian-Doppler Medical Centre in Salzburg, Austria who had received a formal ICD-10 diagnosis in the schizophrenia spectrum group (F20) or the schizoaffective disorders spectrum group (F25). Matching controls were recruited via announcements through the Uiversity of Salzburg mailing list.

Ethics oversight	Recruitment and testing of the INHOUSE sample was approved by the Ethics Board of the University of Salzburg.

Note that full information on the approval of the study protocol must also be provided in the manuscript.

Magnetic resonance imaging

Experimental design

Design type	resting state
Design specifications	resting state
Behavioral performance measures	resting state fMRI was collected, hence no behavioural performance measures were recorded.

Acquisition

Imaging type(s)	functional
Field strength	3
Sequence & imaging parameters	INHOUSE: 32-channel head coil. Functional images were acquired with a T2*-weighted gradient echo EPI sequence (TR 2,250 ms, TE 30 ms, matrix 64 mm × 64 mm, FOV 192 mm, flip angle 70°). Thirty-six slices with a slice thickness of 3 mm and a slice gap of 0.3 mm were acquired within the TR. Scanning was completed over two sessions with 321 scans per session. Finally, a gradient echo field map (TR 488 ms, TE 1 = 4.49 ms, TE 2 = 6.95 ms) and a high-resolution (1 mm × 1 mm × 1 mm) structural scan with a T1-weighted MPRAGE sequence were also acquired.<br>COBRE: A multi-echo MPRAGE (MEMPR) sequence was ran with the following parameters: TR/TE/TI = 2530/[1.64, 3.5, 5.36, 7.22, 9.08]/900 ms, flip angle = 7°, FOV = 256x256 mm, Slab thickness = 176 mm, Matrix = 256x256x176, Voxel size =1x1x1 mm, Number of echos = 5, Pixel bandwidth =650 Hz, Total scan time = 6 min. Resting state data was collected with single-shot full k-space echo-planar imaging (EPI) with ramp sampling correction using the anterior-to-posterior commissural line as a reference (TR: 2 s, TE: 29 ms, matrix size: 64x64, 32 slices, voxel size: 3x3x4 mm3).<br>UCLANP: Functional MRI data were collected with a T2*-weighted echo planar imaging (EPI) sequence with the following parameters: slice thickness = 4mm, 34 slices, TR=2s, TE=30ms, flip angle=90°, matrix=64 × 64, FOV=192mm. A T1-weighted high-resolution anatomical scan (MPRAGE) was collected with the following parameter: slice thickness = 1mm, 176 slices, TR=1.9s, TE=2.26ms, matrix=256 x 256, FOV=250mm.
Area of acquisition	Whole brain scan

Diffusion MRI ☐ Used ☒ Not used

Preprocessing

Preprocessing software	SPM12
Normalization	Linear normalization
Normalization template	MNI
Noise and artifact removal	Motion correction was performed using ICA-AROMA (Pruim et al., 2015; http://fsl.fmrib.ox.ac.uk/fsl/fslwiki/OtherSoftware), and the resulting non-aggressively corrected resting state time series were used for computing the INT.
Volume censoring	No volume censoring was used, beyond motion correction with ICA-AROMA.

Statistical modeling & inference

Model type and settings	Mass univariate with fixed effects was used to identify ROIs. An autocorrelation function was then used to estimate the Intrinstic Neural Timescales (INT) from each participant and ROI.
Effect(s) tested	We tested for group differences in INT within each of the three samples (INHOUSE, COBRE and UCLANP) and between the two experimental groups: patients v. controls.

Specify type of analysis: ☐ Whole brain ☐ ROI-based ☒ Both

Anatomical location(s)	Functional ROIs based on whole-level group differences in the COBRE dataset were defined. Activations were saved as ROIs using Marsbar from SPM12.
Statistic type for inference (See Eklund et al. 2016)	Voxel-wise activations were used to identify ROIs.
Correction	FDR

Models & analysis

n/a	Involved in the study
☒	☐ Functional and/or effective connectivity
☒	☐ Graph analysis
☒	☐ Multivariate modeling or predictive analysis
